# Determine the impact of Emotive Intelligent Spaces on children’s behavioural and cognitive outcomes

**DOI:** 10.1080/2331186x.2023.2281850

**Published:** 2023-11-18

**Authors:** Shiyi Chen, Minyoung Cerruti, Mona Ghandi, Ling-Ling Tsao, Rebecca Sermeno

**Affiliations:** 1 Margaret Ritchie School of Family and Consumer Sciences, College of Agriculture and Life Sciences, University of Idaho, Moscow, ID, USA.; 2 School of Design and Construction, Voiland College of Engineering and Architecture, Washington State University, Pullman, Washington, USA.

**Keywords:** Child Development, Early Childhood, Educational Psychology, colour, intervention, working memory, self-regulation

## Abstract

This study aims to investigate the impact of a novel environmental intervention—Emotive Intelligent Spaces (EIS) on young children’s self-regulation and working memory using a single-subject reversal design (ABAB). EIS is a semi-private space with coloured lights that could adapt to each child’s preferred colour based on the child’s self-reported emotional state. A total of 29 three-to-seven-year-old participants completed the experiment from fall 2020 to summer 2021. Self-regulation was measured by the Head-Toes-Knees-Shoulders task; working memory was measured by the Woodcock-Johnson Numbers Reversed subset. Children’s age was controlled as a covariate. Descriptive statistics indicated that the group means of self-regulation scores were higher in the intervention conditions. However, the group means of working memory scores were lower in the intervention conditions. We conducted repeated measure ANCOVA for the main analysis, and results showed no statistically significant differences in children’s self-regulation and working memory scores between baseline and intervention conditions. It is recommended that future studies should take the illuminance level into consideration of the intervention effect. Further, our study implies that avoiding visual overstimulation in the classroom (e.g. heavily decorated walls) may create an optimal level of visual arousal and promote focused attention.

## 1. Introduction

This study aims to examine the effect of an environmental intervention on young children’s self-regulation and working memory—two cognitive skills that are related to future success in schools and life (e.g., Best & Miller, 2010; McClelland et al., 2014; Rashedi et al., 2021). Self-regulation refers to the ability to control one’s impulses in order to execute goal-oriented behaviour (Vohs & Baumeister, 2004). Research has linked self-regulation to school performance and later developmental outcomes. For instance, children with strong self-regulation skills are more likely to have better academic achievements (Rashedi et al., 2021), graduate from college (McClelland et al., 2013), and have better behavioural and mental health (Moffitt et al., 2011).

Working memory is a critical cognitive process that underpins self-regulation. Working memory temporarily retains and manipulates a small amount of information relevant to problem-solving (Cowan, 2016). To regulate one’s own behaviour, mental representations of goals or rules need to be held in working memory to allow one to monitor and control behaviours accordingly (Zelazo, 2015). Like self-regulation, working memory measured during early childhood predicted children’s later academic achievements such as mathematics and literacy outcomes in elementary schools (Alloway & Alloway, 2010; Swayze & Dexter, 2018). Developmental research shows that early childhood is a critical period for nurturing self-regulation and working memory through daily lives and intervention; failure to do so may lead to challenges in formal school settings (Berkman et al., 2011; Röthlisberger et al., 2013).

Common approaches to enhance self-regulation and working memory include individualized, systematic interventions administered by human or computer programs, which can be expensive and time-consuming (Graziano & Hart, 2016; Judd et al., 2021). Therefore, we created an environmental intervention (i.e., Emotive Intelligent Spaces [EIS]) that is lower-cost than human-mediated intervention. EIS aims to enhance focused attention and cognitive task performance by maintaining an intermediate level of environmental arousal. The purpose of this study is to determine the effect of the EIS intervention on young children’s self-regulation and working memory-two crucial behavioural and cognitive skills related to future development and academic achievement (Rashedi et al., 2021; Swayze & Dexter, 2018). In the following section, we provide an overview of self-regulation, working memory, and the EIS intervention.

### 1.1. Self-regulation

Self-regulation describes an individual’s ability to manage cognition and emotion to take goal-directed action (Rosanbalm & Murray, 2017; Sameroff, 2010; Tominey & McClelland, 2011). Research shows that various levels of self-regulation skills demonstrated during early childhood predict a multitude of short- and long-term outcomes such as school readiness, academic achievement in primary school, and ability to cope with stress and substance use later in life (McClelland et al., 2013). Self-regulation is supported by three overlapping cognitive functions: working memory, mental flexibility, and inhibitory control (Diamond, 2013). Although those skills are far from maturing during early childhood, children can develop those skills with adults’ guidance (Robson et al., 2020). Children with good self-regulation skills are able to manage their own behaviour, emotion, and attention to achieve a goal (Rimm-Kaufman et al., 2000).

Self-regulation is a more robust predictor of successful learning in schools than content knowledge like literacy or mathematics (Blair & Razza, 2007; McClelland et al., 2014; Rashedi et al., 2021). Therefore, early childhood professionals have become interested in strategies that improve self-regulation (e.g., modeling, role-playing) during this period of rapid brain development (Diamond, 2013). Early childhood professionals need to be mindful and intentional when teaching self-regulation through daily routines and activities. Some popular strategies include building warm relationships with each child, responding to children’s needs with warmth in stressful situations, and working closely with parents (Pahigiannis et al., 2019). With limited interventional options, mindfulness-based interventions (e.g., yoga) led by a trained coach (e.g., Rashedi et al., 2021; Viglas & Perlman, 2018) seem to become popular in early childhood settings. However, these self-regulation strategies and interventions heavily rely on human interactions and can be expensive and time-consuming (Pandey et al., 2018). Thus, there is a need for children’s self-regulation interventions with less dependency on adult support, such as environment-assisted co-regulation interventions.

### 1.2. Working memory

Working memory is the ability to temporarily store and manipulate information to execute complex cognitive tasks (Forsberg et al., 2021). It is a dynamic cognitive function that modulates task-relevant information for long-term storage or problem-solving (Sepp et al., 2019). Working memory is different from other types of memory such as sensory memory (i.e., a type of brief memory that contains impressions from senses such as vision and auditory information) and long-term memory (Cowan, 2016; i.e., a type of memory that stores a large amount of information for a long period of time). Further categorization of working memory includes visual-spatial and auditory/verbal memory (Miller, 2022), corresponding to the three functional components of working memory: phonological loop (processing verbal information), visuospatial sketchpad (processing visual information), and central executive (i.e., attention control).

Like self-regulation, working memory is evidently linked to school achievement (Fitzpatrick & Pagani, 2012; Swayze & Dexter, 2018). For example, Alloway and Alloway (2010) found that children’s verbal working memory at five years of age predicted their literacy and numeracy achievement six years later. Results from a large-scale longitudinal study suggest that preschool children’s visual-spatial working memory was associated with their kindergarten school readiness measured as classroom engagement, number knowledge, and receptive vocabulary (Fitzpatrick & Pagani, 2012). In a more recent study by Swayze and Dexter (2018), preschool children’s verbal working memory is strongly associated with their kindergarten readiness measure in five content-specific areas (colours, letters, numbers/counting, size/comparison, and shapes).

Whether working memory is changeable via education and training remains debatable. Researchers created intervention and training programs that have been shown to improve working memory (e.g., Kroesbergen et al., 2014; Roberts et al., 2016; Rowe et al., 2019); however, the effect of these programs tend to be acute and are not transferable (Chen & McDunn, 2022; Sala & Gobet, 2020). Despite the limited malleability of working memory, working memory capacity could be supported or hindered by internal and external factors such as emotions and environmental stimuli (Paas & van Merriënboer, 2020). For instance, a meta-analysis shows that working memory performance was negatively related to self-reported anxiety levels (Moran, 2016). Shipstead and colleagues (Shipstead et al., 2015) reported an association between attention and working memory capacity, indicating a negative relation between environmental distraction and working memory (Paas & van Merriënboer, 2020). Thus, it is plausible to assume that environmental interventions could potentially support working memory.

### 1.3. The environment and behavioural and cognitive outcomes

As mentioned above, using the environment to help children co-regulate their behaviours and facilitate their working memory performance could be an easy-to-implement and cost-effective intervention. In particular, the colour of the physical environment can impact individuals’ behavioural and cognitive outcomes (Castell et al., 2017; Engelbrecht, 2003; Ghandi et al., 2021; Read, 2019; Zhu et al., 2019). For instance, research shows improved alertness and self-regulation in adults after daytime exposure to higher correlated colour temperature lighting (i.e., natural white light) (Steidle & Werth, 2014). Similarly, elementary school-aged children in a higher correlated colour temperature condition exhibit better-sustained attention as compared to those in a lower correlated colour temperature lighting condition (i.e., dim yellow light) (Pulay et al., 2018). Other studies also indicate environmental colour’s effect on children’s creativity and detail-orientated tasks (Mehta & Zhu, 2009), cooperative behaviour (Read, 2019), and emotion (Zentner, 2001). Although certain colour-emotion associations (e.g., red is associated with excitement or anger) are relatively consistent across different populations, the emotional interpretations of colour can vary depending on accumulating common experiences and gender differences (Hanada, 2018). For example, people from the U.S. associate the yellow-green colour with disgust, whereas the same colour is interpreted as relaxation by Kenyans (Kaya & Epps, 2004). In another study, seven-to-eight years old girls rate pink high on happiness whereas boys of the same age rate pink low on happiness (Pope et al., 2012).

Our environmental intervention, Emotive Intelligent Spaces (EIS), is a semi-private space with coloured lights that can adapt to each child’s preferred colour based on the child’s self-reported emotional state. The design of EIS is guided by two theories pertaining to environment-behaviour perspectives. First, the Yerkes-Dodson law (Eysenck, 1982) proposes that an optimal level of performance is associated with an intermediate level of environmental arousal (Fisk et al., 2018). Second, Attention Restoration Theory (Kaplan & Berman, 2010) states that an individual’s ability to direct attention is supported by environmental features that elicit cognitive restoration and are compatible with individual preferences. Based on these theories, the three premises of the EIS are (1) coloured lighting as an environmental stimulus can positively or negatively contribute the emotional arousal and attention control (Pulay et al., 2018), (2) emotional arousal can modulate an individual’s task performance (Turkileri et al., 2021), and (3) emotion-colour association may differ between individuals based on cultures, context, and interpretations (e.g., blue could represent calmness or sadness; Hanada, 2018). Thus, the EIS intends to restore attention and improve children’s behavioural and cognitive outcomes by changing the colour of the environment based on the child’s preferred colour that corresponds to the individual’s emotional arousal as supported by the theories.

### 1.4. Empirical gaps

The proposed project bridges the empirical gap regarding the effect of environmental colour on children’s behavioural and cognitive outcomes. Research studies on the association between colours and cognitive and affective outcomes have been conducted extensively on adults’ moods and cognitive performances (Wilms & Oberfeld, 2018). For example, red is associated with higher anxiety levels but yields better detail-oriented task completion among college students (Read, 2019); green is linked to lower stress responses and promotes creativity in adults (Elliot, 2015). However, only a handful of research on a similar topic was conducted with children. For instance, red is linked with children’s excitement, alertness, and anger (Boyatzis & Varghese, 1993; Karp & Karp, 2001); blue is associated with calmness and lower body temperature (Engelbrecht, 2003); and grey is linked to negative emotions, particularly in girls (Boyatzis & Varghese, 1993). Besides, children’s perceived colour-emotion associations can vary by culture and gender (Pope et al., 2012). Very little is known about the effect of children-preferred colour on their behavioural and cognitive outcomes. Additionally, typical early childhood interventions rely on trained interventionists, which could be costly (Pahigiannis et al., 2019; Rashedi et al., 2021). Thus, there is a need for cost-effective interventions that capitalize on the environmental features. To fill the empirical gaps, our study aims to determine the effect of EIS—an adaptive environmental intervention that uses young children’s preferred colour to improve self-regulation and working memory.

### 1.5. Research questions and hypotheses

To bridge the gaps found in the literature, this study seeks to answer two research questions: 1) Whether being in the EIS environment with personalized coloured light has an impact on children’s self-regulation? 2) Whether being in the EIS environment with personalized coloured lights has an impact on children’s working memory? Children’s age was controlled as a covariate in both research questions. We hypothesized that children’s working memory and self-regulation scores would be higher in the intervention condition (i.e., with the EIS coloured lighting) than in the baseline condition (i.e., typical fluorescent lighting).

## 2. Materials and method

### 2.1. Methodology

Single subject experimental design is a research method that provides strong empirical evidence of intervention effectiveness, in particular for its real-life applications (Horner et al., 2005). The single-subject reversal design compares participants with their own performance across conditions to establish a functional relationship between outcomes and the intervention (Kazdin, 2011). Thus, this study adopted a rigorous, systematic method of single-subject reversal design (i.e., ABAB design) to document and replicate a functional and causal relationship between independent (i.e., coloured lights) and dependent (i.e., self-regulation, working memory) variables (Horner et al., 2005). The intervention in this study employs an ABAB research design (A=baseline with no intervention, B=intervention with coloured lights), in which, the first A-B conditions were repeated to establish the evidence.

### 2.2. Participants and setting

Upon the Institutional Review Board’s approval, research study flyers were distributed via emails, community bulletin board posting, and social media outlets. Based on the prior power analysis, we recruited 41 typically developing children from several local early childhood programs in northern Idaho and eastern Washington in fall 2020 and spring 2021. A total of 39 participants completed Day 1 data collection (i.e., screening), among which 35 participants also completed Day 2 data collection (i.e., intervention). Six out of the 35 participants were excluded because they scored zero on all measurements. The final sample size used for the data analysis was 29 (see Table 1). Participants were predominately White, female, with an average age of 66 months (5.5 years) in a range from 47 to 95 months.

The experiment was conducted at the lead author’s university in a research laboratory in Spring and Summer 2021. The research lab was 20 feet wide, 45 feet long, and 9 feet and 9 inches high. The lab had white walls, light grey vinyl flooring, and fluorescent lighting. The lab contained two large outside windows, which were covered up with blinds to fully control the lighting conditions during the experiment. Basic illuminance was offered in the lab through two large ceiling luminaires (providing an average of 300 lux). The EIS panels were securely installed in a corner of the research lab, and a six-foot-wide round child table and two child chairs were placed inside the EIS.

### 2.3. EIS design

**Figure 1.**
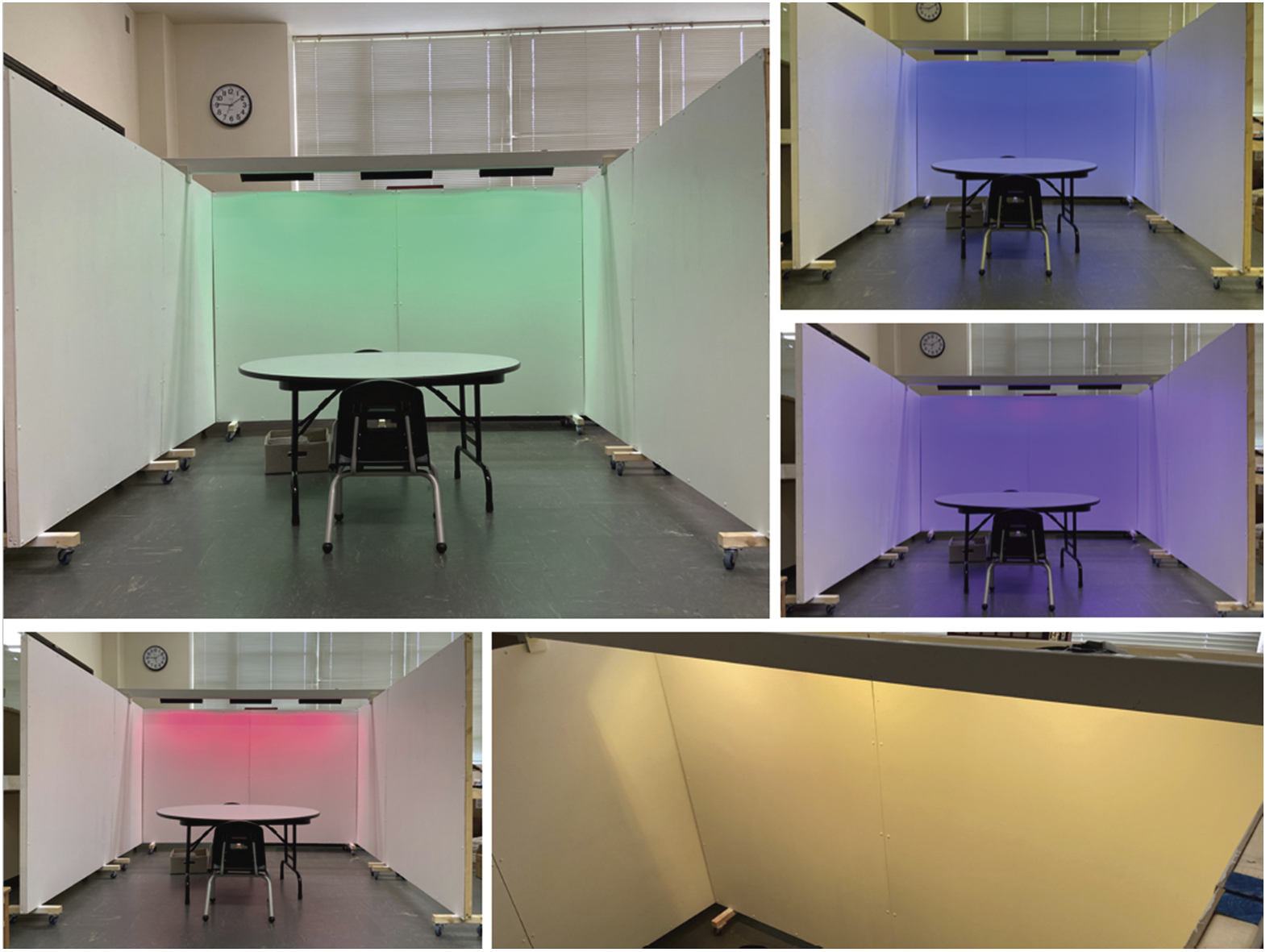
Emotive Intelligent Spaces. *Note*. This figure demonstrates the EIS space and the coloured lighting changes in the intervention condition.

The EIS design consisted of six wooden panels (Figure 1). Each panel was three feet wide by five feet high and was made from double layers of wood in white. Three LED light boxes (17.32 × 4.13 × 2.56 inches) were mounted above the panels and diffused the coloured lights on the surface of the panels facing the children. The colour of the LED lights was controlled by a smartphone application, which allowed the research assistants to change three LED light boxes simultaneously based on the participants’ self-reported emotional states. The hue and brightness of the coloured lights were adjusted to match the children’s preferred colours indicated by the colour-emotion association assessment (Day 1) prior to the experiment (see Day 1 data collection described in a later section).

### 2.4. Measurements

The Head-Toe-Knees-Shoulders task (HTKS), a well-validated directed assessment, was used to assess children’s behavioural self-regulation (i.e., ability to focus on instruction and process the current trial while keeping the rule in mind) (McClelland et al., 2014). There are a total of 30 test items in three sections. Sections 1 and 2 include a combination of two body parts (e.g., head/toes; knees/shoulders), and participants are asked to do the opposite of the experimenter’s commands. Section 3 includes a rule switch of combinations of four body parts. Scores range from 0 (incorrect), 1 (self-correct), to 2 (correct) for each item. HTKS has strong inter-rater reliability (*κ* = 0.90; McClelland et al., 2014) and high reliability (*α* = .94; McClelland et al., 2014). Two parallel forms (form A and B) were used in alternating conditions to prevent the participants from building familiarity with the test content.

Children’s working memory was measured by the Woodcock-Johnson-IV (WJ-IV) cognitive assessment Numbers Reversed subset (Schrank et al., 2014). This task requires participants to hold a series of presented numbers (e.g., 3, 8, 1) in short-term memory while reversing the sequence (e.g., 1, 8, 3). The number sequence started with a two-digit combination and gradually increases. The task would stop after participants produce five consecutive incorrect answers. Each item was scored as 1 (correct) and 0 (incorrect). Like the HTKS, we used two parallel forms in alternating conditions.

### 2.5. Data collection procedure

**Figure 2.**
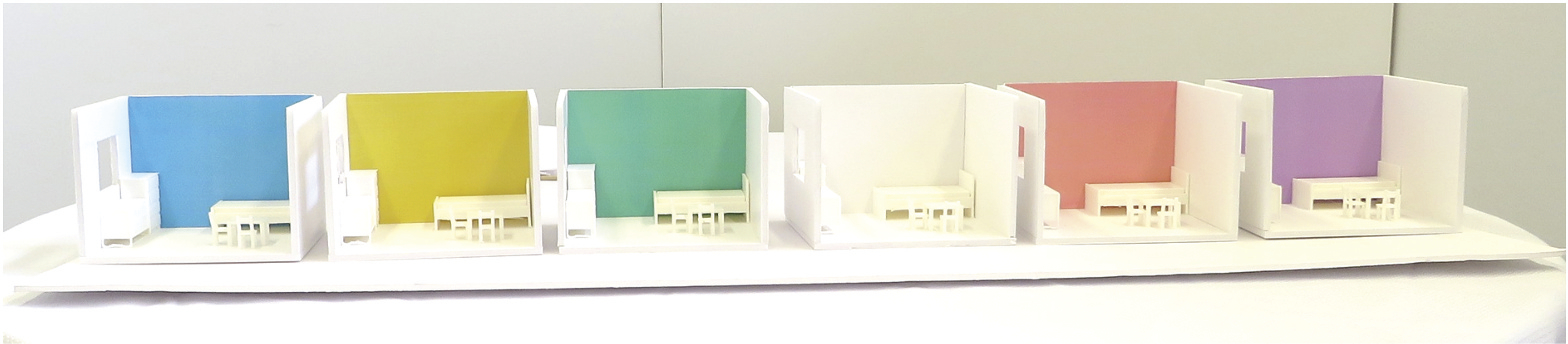
Child room models for the colour-emotion association test. *Note*. These child room models with colourful walls were used in Day 1 data collection, where children were presented with

Given young children’s short attention span and the length of the intervention conditions, data were collected on two days. As the EIS required colour perceptions, Day 1 was designed to screen participants for colour deficiency, identify participants’ preferred colours under three emotional statuses (happy, sad, angry), and acclimate participants to the experiment setting. Day 1 data collection took place in March and April 2021, during which trained research assistants administered the Ishihara colour deficiency test (Ishihara, 2010) and the colour-emotion association questionnaire in the EIS environment with basic ceiling lights and no coloured lights on. Given young children’s limited language ability, we constructed a scenario using a dog puppet “Toto” and six physical models of a typical toddler’s bedroom on a scale of 1’ = 1/2” to give children a concrete representation of colour-emotion association (Figure 2). Each physical model had different colours: red (5 R–6/8), yellow (5Y–7/8), green (5 G–6/8), blue (5B–6/8), purple (5P–6/8), and white. The colours were identified from one of the authors’ pilot study as the most preferred colours by young children from each of five hue families from the Munsell colour system. Six physical models were displayed in a row and the arrangement order of the models was randomized for each child using a random number table. The research assistants told children three stories representing three basic emotions using the puppet and a small gift box (i.e., Happy—Toto received a gift from his grandma; Sad—Toto lost his toy; Angry—Toto’s sister took the toy away). Immediately after each scenario, the research assistants asked children to identify colours that they perceived to be associated with happiness in the “Happy” scenario (“Which room should Toto go to play with the toy?”) and to identify colours that children thought will restore Toto’s mood to “Happy” in the “Sad” or “Angry” scenario (“Which room should Toto go to make him feel better?”). The goal of these colour preference questions was to identify each child’s self-reported colours that might help them maintain the optimal emotional arousal defined as the Happy condition. Children’s colour preference under each scenario was recorded as their personalized colour-emotion association and was used in Day 2 data collection.

**Figure 3.**
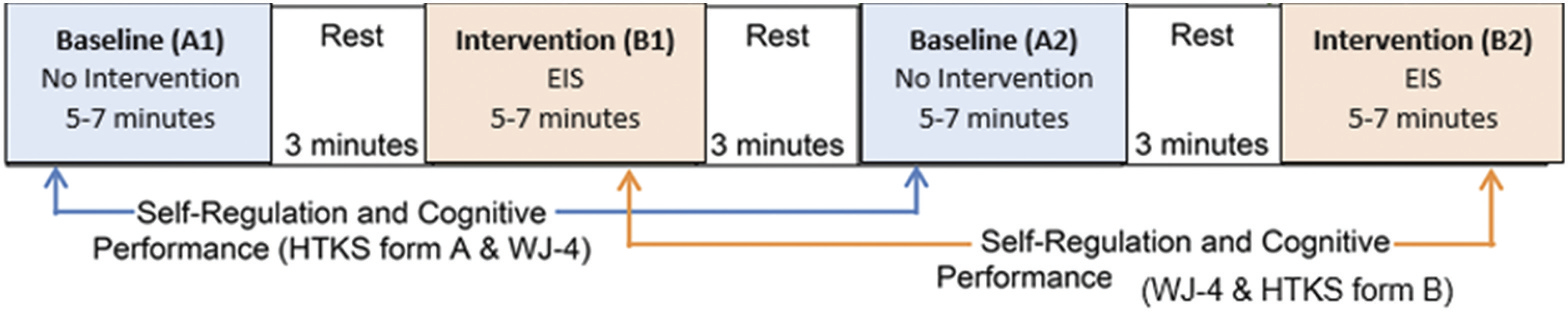
Experiment procedure. *Note*. A = baseline condition; B = intervention condition; HTKS = Head-Toes-Knees-Shoulders; WJ-4 = Woodcock and Johnson IV

The experiment took place on Day 2 data collection in June 2021. Participants were instructed to sit at a child-size table in the EIS environment facing the panels with coloured lights (Coloured lights were off during baseline conditions). As Figure 3 indicates, the research assistants administered the self-regulation and working memory assessments under two cycles of both baseline and intervention conditions using two parallel versions of the same assessments in alternating conditions (e.g., assessment version I in the A baseline condition; assessment version II in the B intervention condition). This is to minimize children’s testing fatigue and item memorization. The self-regulation task was video recorded and two trained research assistants scored all video recordings in order to calculate inter-rater reliability that adjusts for an expected agreement based solely on chance (Cohen, 1960). Cohen’s kappa showed satisfactory inter-rater reliability (*κ* = .89). There were three five-minute breaks between conditions to avoid fatigue. Before each intervention condition, children were asked to self-report their emotional state by selecting one of the three emotional faces displayed on the table: happy, sad, or angry. The research assistants then turned on the participant’s preferred coloured lighting based on the participant’s reported emotional state and his/her colour-emotion associated reported in Day 1 data collection. For instance, if a child self-reported feeling sad, the research assistant then turned on the coloured light that would make that child feel better under a sad condition, as the child identified in the Day 1 data collection. On average, a Day 2 data collection lasted 60–70 minutes. Parents received a digital gift card as a thank-you for their participation in the study at the end of their data collection.

### 2.6. Data analysis strategy

Repeated-measures analysis of covariance (ANCOVA; Bakdash & Marusich, 2017) was used to determine the mean differences in children’s self-regulation and working memory scores under each condition (ABAB) as within-subject factors. ANOVA is a statistical method that analyses the statistical difference between three or more group means while controlling for the effect of at least one confounding variable (Rutherford, 2000). Age was added as a covariate in order to control the effect of maturation on children’s self-regulation and working memory. The effect size was estimated using partial-*η*^*2*^ statistics (small: *η*^*2*^ ≥0.01; medium: *η*^*2*^ ≥0.06; large: *η*^*2*^ ≥0.14) to measure the magnitude of the observed effect of the intervention (Cohen, 2013). Data analysis was conducted using SPSS.

## 3. Results

Six out of the 35 participants who completed the experiment were excluded because they scored zero on all measurements. A total of 29 children were included in the main data analysis. Descriptive statistics, box plots and bar graphs indicated that there was no outlier and data were normally distributed. Descriptive analysis (Table 2) indicated that the group average self-regulation scores as measured by HTSK were higher in the intervention conditions than in the baseline conditions (*M*_*A1*_ = 27.60, *M*_*B1*_ = 32.03, *M*_*A2*_ = 29.67, *M*_*B2*_ = 30.27). However, the group means of working memory scores as measured by WJ-IV-Numbers Reversed were lower in the intervention conditions than in the baseline conditions (*M*_*A1*_ = 4.67, *M*_*B1*_ = 4.43, *M*_*A2*_ = 4.90, *M*_*B2*_ = 4.53).

**Figure 4.**
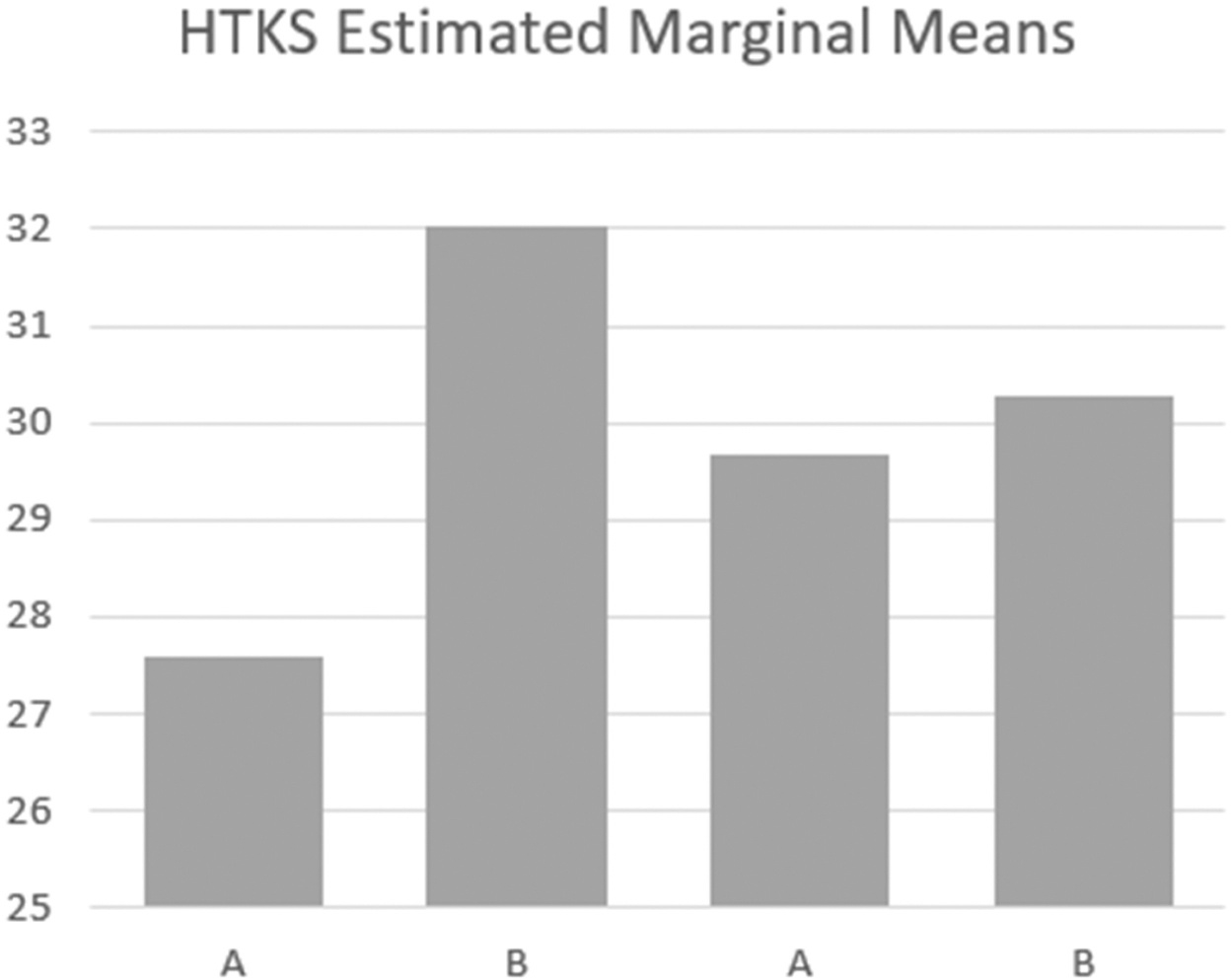
ANCOVA analysis results – self-regulation. *Note*. A = baseline condition; B = intervention condition; HTKS = Head-Toes-Knees-Shoulders

**Figure 5.**
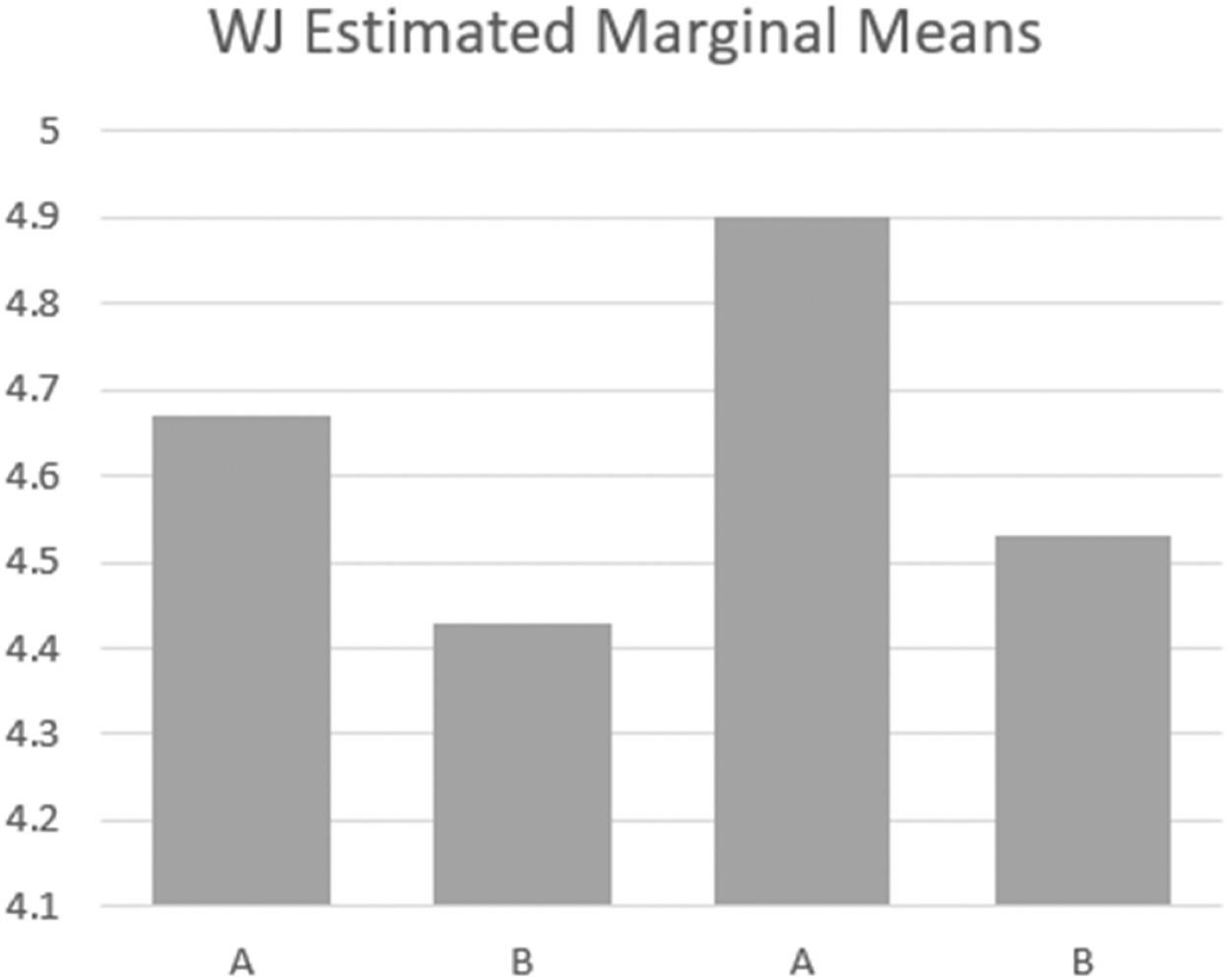
ANCOVA analysis results – working memory. *Note*. A = baseline condition; B = intervention condition; WJ = Woodcock Johnson (Numbers Reversed subset).

We conducted repeated-measures ANCOVA to answer two proposed research questions: 1) Whether being in the EIS environment with personalized coloured light has an impact on children’s self-regulation? 2) Whether being in the EIS environment with personalized coloured lights has an impact on children’s working memory? Contrary to our hypotheses, results indicated that children’s HTKS scores did not statistically differ between baseline and intervention conditions, controlling for children’s age (*F*(3, 28) = .46; *p* = .71). Partial eta square suggested a small effect size (*η*^2^= .02). Similarly, children’s working memory did not statistically differ between baseline and intervention conditions, controlling for age (*F*(3, 28) = .71; *p* = .55) with a small effect size (*η*^*2*^=.03). Overall, ANCOVA analysis suggested that children’s self-regulation and working memory did not meaningfully change between baseline and intervention conditions; the score differences between baseline and intervention conditions were likely due to chance (see Figures 4 and 5).

## 4. Discussion

The purpose of this study is to determine the effect of EIS on children’s behavioural and cognitive outcomes which are measured as behavioural self-regulation and verbal working memory, using a single-subject reversal design. We hypothesized that children would score higher on behavioural self-regulation and verbal working memory assessment in a personalized coloured lighting environment than in a typical fluorescent lighting environment. However, results from a series of ANCOVA suggested that EIS did not significantly impact children’s behavioural or cognitive outcomes.

### 4.1. EIS’ impact on self-regulation

Results showed no statistical differences in children’s behavioural self-regulation scores between baseline and intervention conditions, but the group means in the intervention conditions were higher than the baseline conditions. The descriptive finding contradicts previous research on lighting and self-regulation, where adult participants scored higher under bright natural white light (Steidle & Werth, 2014). However, our finding should be interpreted with extreme care because the insignificant ANCOVA results indicated that the group mean differences in our study could be due to chance.

There may be four plausible explanations for the null results: first, it was possible that some children might have memorized certain HTKS and WJ-IV Number Reversed items after the first few rounds of testing, despite the use of parallel forms. If this was the case, children’s self-regulation and working memory scores did not accurately reflect the intervention effect (Thomson & Oppenheimer, 2016). Second, the dimness of the data collection room may contribute to a lack of self-awareness and alertness as supported by Steidle and Werth’s study (Steidle & Werth, 2014), in which, college-aged adults demonstrated an improvement in self-regulation in a bright light condition compared to a dim light condition. In comparison, we purposefully covered outward-facing windows with blinds in the laboratory for the coloured lighting to wash evenly on the EIS panels with no interruption by sunlight through the windows, which resulted in dimmer baseline conditions and may negatively impact children’s self-regulation in the baseline conditions. Unfortunately, we were not able to find similar studies conducted with children. Third, the color intensity of EIS’ lighting could be too strong rather than intermediate. According to our theoretical frameworks of Yerkes-Dodson Law (Eysenck, 1982) and Attention Restoration Theory (Kaplan & Berman, 2010), heightened arousal may compromise children’s task performance and impede their ability to direct attention to the task. The potential overstimulation effect of the lights is detrimental particularly to young children because their ability to sustain focused attention is very limited (Cowan, 2016). Lastly, the ABAB design may be too long for young children’s attention span, especially for boys (Pham, 2016). Although we prepared two parallel versions of the same assessment, being tested four times on similar tasks within an hour could potentially exhaust young children’s attention.

### 4.2. EIS’s impact on working memory

Contrary to our hypothesis, children’s verbal working memory scores in the intervention conditions did not differ significantly from the baseline conditions. Surprisingly, the group means of working memory scores in the intervention conditions were lower than the ones in the baseline conditions, which is the opposite of the self-regulation group means. (Table 2). Considering that the participants only received verbal cues in the working memory tasks but received both verbal and visual cues in the self-regulation task, coloured lighting, as a potential distractor, may have a larger negative impact on the working memory task by increasing the cognitive load (Paivio, 2014). Again, the group means differences in our study have very limited interpretability due to the statistically insignificant ANCOVA results.

Given that working memory is subject to participants’ attention (Shipstead et al., 2015) and that young children’s working memory capacity and attention span are far from maturing (Cowan, 2016), it is possible that the lighting could be a salient distraction for the verbal working memory task. Tying back to one of our theoretical frameworks—the Yerkes—Dodson Law (Eysenck, 1982), the coloured lighting might evoke a high-level of environmental arousal in young children, therefore did not elicit optimal task performance. This speculation is in line with Rodrigues and Pandeirada (2018), in which children (age = 8–12 years old) scored poorer on the working memory and inhibitory control tasks in an environment with high-load visual stimulation than in an environment with natural lighting (i.e., ceiling light plus sunlight) and no decorations. In another study conducted with high schoolers, there were no statistical differences in participants’ cognitive performance between different colourful environments (Castell et al., 2017). However, some research on the effect of specific colours, rather than colours of participants’ preference, yielded significant findings (Engelbrecht, 2003; Zhu et al., 2019). For example, Zhu et al. (2019) report an association between low warm lighting (i.e., dim, yellow lights) and poorer cognitive performance. In contrast, they found that high cool lighting (i.e., bright white light) was linked with improved memory recall tasks.

Although this study did not yield any statistically significant findings, it has several potential implications in children’s classroom settings. The first implication is to present both auditory and visual information to enhance learning. In our study, participants received both auditory and visual cues in the HTKS tasks but only received auditory cues in the WJ-IV tasks. We found that the group average HTKS score was higher in the intervention conditions than in the baseline conditions, however, it was the opposite for the WJ-IV Numbers Reversed. It is possible that the combination of audio and visual cues enhanced participants’ memorization. This is consistent with the dual-coding model, which states that brains encode audio and visual information differently, therefore, learners are more likely to remember information that is presented and rehearsed via both auditory and visual means (Paivio, 2014). The second plausible implication is to improve children’s attention by adjusting the level of environmental arousal in the classroom in relation to the type of activities. Our study showed that the coloured lighting might not function as an intermediate stimulus for cognitive performance (i.e., working memory task); rather, it could be an overstimulation (e.g., visual noise by colour change) when being combined with other visual overstimulation (e.g., materials and decorations) present in typical classroom settings. For instance, it is a common practice in early childhood classrooms in the U.S. to decorate the wall and floor with as many colourful learning materials (e.g., alphabet chart and numbers rug) as possible (Godwin et al., 2022). However, these busy visual stimuli might become a source of negative distraction, which in turn impedes children’s learning (Fisher et al., 2014). Certain types of early childhood education programs such as Reggio Emilia and Montessori schools have adopted a minimalist classroom design with neutral colours and simple classroom decorations (Aljabreen, 2020). Perhaps early educators could consider adjusting the amount of visual stimulation in the classroom to optimize the level of arousal and facilitate learning in accordance with types of activities or tasks.

### 4.3. Limitations and future directions

Our study has four main limitations. First, six participants withdrew from the study and another six participants were not included in the analysis due to unusable data. The final sample size (*N* = 29) was smaller than the power analysis estimation (*N* = 40); therefore, the analysis was underpowered, which lower the chance of detecting a statistically significant result if the effect was present (Fan, 2001). Second, completing two intervention and baseline conditions in one sitting seemed to be too long for young children, which might have interfered with the intervention and the quality of the data. Based on research assistants’ observations, a number of children showed signs of boredom after the first baseline and intervention conditions and were reluctant to complete the second baseline and intervention conditions, despite the breaks between conditions and the use of two parallel versions of assessments. This was particularly true for children younger than 48 months old, as they seemed to have great difficulty staying on task after the first two conditions. Third, the assessments were administered immediately after the light was turned on in both intervention conditions without sufficient EIS coloured lighting exposure prior to the testing. We observed that children tended to be very intrigued by the coloured lights, which might have distracted children’s attention and negatively impacted their assessment results. Fourth, we only installed the lights on the wall facing child participants. This lighting environment may not be immersive enough to produce meaningful changes in children’s behavioural and cognitive performances. Fifth, certain coloured lighting (e.g., purple) was somewhat dim. A lighting study found lower cognitive performance in a dim lighting environment (Pulay et al., 2018). Last but not least, our participants performed well on the working memory assessment when comparing their scores with the WJ-IV norm reference, which is understandable given our participants’ high parental education level (Grindal et al., 2016). Therefore, WJ-IV Numbers Reversed subset may not be sensitive enough to discriminate children’s performance in association with the coloured light influence.

For the next step of the research, we plan to improve the study design and test the intervention’s effect with children with hyperactivity disorder (ADHD). Self-regulation deficits are common across some neurodevelopmental disorders such as ADHD (Barkley, 1997). Current non-pharmacological intervention for ADHD includes interventions focusing on ADHD symptoms (e.g., hyperactivity/impulsivity) to improve school performance (e.g., academic or classroom behaviour) and associated difficulties (e.g., social or emotional behaviours). However, these studies yielded mixed results (see Moore et al., 2018 for details). Supporting emotional regulation actually holds the most promise for improving the academic attainment of children with ADHD (Moore et al., 2018, p. 246). It would be of value to examine how the proposed intervention may impact the performance of children with ADHD since limited interventions have been designed to target this area. In our future study, we will give participants 10–20 seconds to rest before testing under the intervention condition. This is to allow them to acclimate to the EIS lighting, therefore, becoming familiar with the intervention environment and diminishing the novel situational effect. We plan to adjust the lighting to keep the illuminance level constant in both baseline and intervention conditions and avoid dim environments. Also, we plan to use an AB or ABA research design instead of ABAB in our future study with children with ADHD. Although the ABAB design yields better control and more rigorous results, our study demonstrated that young children were bored during the second cycle of AB conditions, which might have negatively impacted their assessment scores.

## 5. Conclusion

Emotive Intelligent Space (EIS) aims to restore children’s attention by maintaining an intermediate level of environmental arousal. In this study, we examined the effectiveness of EIS on young children’s behavioural and cognitive performance. Contrary to our hypotheses, we did not find any statistically significant improvements in children’s self-regulation and working memory under the intervention conditions (i.e., with the coloured lighting on). The null results have potential implications for future research and classroom practices. Specifically, we were able to identify several potential experiment design flaws that may be helpful for future research on similar topics. For instance, future studies should avoid dim lighting, and provide sufficient time to allow participants to acclimate to the intervention environment. Also, we recommend using an AB or ABA rather than ABAB experiment design to reduce participants’ boredom and fatigue when data collection has to be completed during one visit. If an ABAB design must be used, the child participants could complete two sets of ABs on separate days. Our study also has potential practical implications. We found that, under potential distraction, children performed better on average in the task where verbal and visual cues were given than in a task where only verbal information was presented. Similar to previous research (Paivio, 2014), our results indicated that children may benefit from learning via both auditory and visual means simultaneously. Further, the results imply a potentially negative effect of too many visual stimuli in the classroom on children’s cognitive performance and self-regulation. As we noted earlier, the average score of working memory was lower in the intervention condition with coloured lighting as compared to the baseline condition (i.e., typical fluorescent lighting). We speculate that this finding might be due to the overly stimulating, instead of an intermediate level of visual arousal, which disrupted rather than enhanced children’s focus. Therefore, teachers may consider simplifying overly decorated classrooms to create an optimal level of visual arousal that promotes focus.

## Figures and Tables

**Table 1.** Demographic information (N_child_ = 29; N_parent_ = 21)

	N	M/percent	SD	Min.	Max.
CHILD					
Gender					
Male	12	40			
Female	17	60			
Age (month)	29	66.29	14.71	47	95
Race/Ethnicity					
Hispanic	3	10	3	10	3
White	23	90			
Asian	2	7			
Bi-racial	1	3			
English as fist language	27	93			
School Placement					
Private Childcare	4	13			
Public Schools	7	25			
Home School	7	25			
Other	11	37			
PARENT					
Age (yrs)	21	34.81	4.31	24	42
Race/Ethnicity					
White	20				
Asian	1				
Degree					
Professional Degree	1				
AA	1				
BA/BS	8				
MA/MS and Above	11				
Marital status					
Single	1				
Married	20				
Household members	29	4.52	1.16	3	8
Household income					
Less than $20,000	3				
$20,000 – $44,999	3				
$45,000–$139,999	14				
$140,000–$149,999	1				

*Note.* AA= Associate Degree; BA/BS= Bachelor’s Degrees; MA/MS = Master’s Degree.

**Table 2.** Descriptive statistics: working memory and self-regulation (*N* = 29)

		M	SD
HTKS	A_1_	27.60	21.43
	B_1_	32.03	22.49
	A_2_	29.67	23.09
	B_2_	30.27	23.26
WJ-IV–Number Reversed	A_1_	4.67	4.28
	B_1_	4.43	4.10
	A_2_	4.90	4.03
	B_2_	4.53	3.86

*Note.* HTKS = Head-Toes-Knees-Shoulders, WJ-IV = Woodcock Johnson-IV, A = baseline condition, B = intervention condition.
